# Sequence–lithofacies paleogeographic evolution and its control on deep and ultra–deep reservoir types: A case study of the Permian Maokou Formation in northeastern Sichuan Basin

**DOI:** 10.1371/journal.pone.0327224

**Published:** 2025-07-01

**Authors:** Xiandong Wang, Wancang Tan, Peng Lu, Ying Wang, Qiang Li, Liang Zhao, Pengfei Niu, Zhengyi Cang, Bao Gong, Jiagang Shen

**Affiliations:** 1 The Exploration & Development Institute of Daqing Oil field Ltd.Company, Daqing, China; 2 College of Earth Science, Northeast Petroleum University, Daqing, China; Wadia Institute of Himalayan Geology, INDIA

## Abstract

The Middle Permian Maokou Formation in northeastern Sichuan Basin has been explored with continuous breakthroughs in recent years, but its sequence–lithofacies paleogeographic characteristics and tectonic–sedimentary evolution process are still unclear. Based on outcrops, well logging and seismic data, this paper focuses on the sequence stratigraphic, lithofacies paleogeography and tectonic–sedimentary evolution of the Maokou Formation in northeastern Sichuan Basin. The results show that two third-order sequences (SQ1, SQ2) are developed in the Middle Permian Maokou Formation. SQ1 can be divided into three fourth-order sequences (SQ1–1, SQ1–2, and SQ1–3). SQ1–1 and SQ1–2 are generally carbonate ramps, SQ1–3 turns to rimmed carbonate platform, and its stratigraphic thickness and sedimentary facies are distributed in NW–SE direction. The succession development of carbonate platform in SQ2 sedimentary period. It is believed that, due to the NE–trending extensional stress generated by the continuous subduction of the Mianlue Ocean and the regional sea level eustacy, the Permian tectonic–sedimentary differentiation in the northeastern Sichuan Basin had begun from the early stage of the Middle Permian and gradually intensified. As a result, the platform–basin dominated deep-water deposits gradually expanded into the basin along SW–NE direction, and evolved into the features of “platform in the south and platform–basin in the north” at the end of the Maokou Formation deposition. As such, large-scale high-energy shoals are developed above the platform region during the SQ1–3 and SQ2 depositional periods, making it a prospect of conventional gas. Potential source rocks are developed in the platform-basin region, which is a potential prospect of unconventional oil and gas. The results suggest that the tectonic–sedimentary differentiation induced by the continuous subduction of Mianlue Ocean makes the Maokou Formation an ideal reservoir for both conventional and unconventional hydrocarbon.

## Introduction

Deep and ultra–deep marine carbonate rocks are rich in oil and gas resources and have great exploration potential. In recent years, they have been one of the most important domains for increasing reserves and production of onshore oil and gas in China [1–3]. China’s marine deep and ultra-deep oil and gas exploration began in 1976. After decades of long-term exploration, several deep and ultra-deep large and extra-large oil and gas fields have been discovered in the Sichuan Basin and the Tarim Basin [4,5]. Specicially, the deep and ultra–deep oil and gas resources in the Tarim Basin are 34.5 × 10^8^t and 5.98 × 10^12^m^3^ respectively, and a large marine deep and ultra-deep oil province with reserves scale of 10 × 10^8^t has been discovered in the Fuman–Shunbei region [6,7]. In the Sichuan Basin, the most prominent ones are the large and super–large gas fields with reserves scale of 100 billion cubic meters formed in the gas reservoirs of the Sinian Dengying Formation and Cambrian Longwangmiao Formation in the central part and the Permian Changxing Formation gas reservoirs in the northeastern part [8–12]. The analysis shows that the formation of the above oil and gas fields is closely related to the evolution of platform–trough under the tectonic–sedimentary differentiation [13–15]. Moreover, recent breakthroughs in unconventional oil and gas exploration in aulacogens [16,17], which have become favorable exploration zones for both conventional and unconventional oil and gas.

The Middle Permian Maokou Formation, as one of the important strata for marine oil and gas exploration in the Sichuan Basin, has been explored for more than 60 years. Early exploration drillings and researches mainly focused on the southern and central parts of the Sichuan Basin, with the main goal of finding fractured and vuggy limestones, and achieved rich results [18–20]. In recent years, with the continuous accumulation of exploration data in the basin and the discovery of siliceous radiolarian–bearing black marl representing deep–water facies in the Maokou Formation of the Xibeixiang section, as well as several high–yield industrial gas flow wells in the shoal dolomite reservoir of the Maokou Formation in the central-north part of the basin [15], the understanding of the sedimentary pattern of the Maokou Formation has gradually changed from the early stable platform to the “platform in the south and rift in the north” [21–26]. Afterwards, the research on the deep-water trough of the Maokou Formation in northeastern Sichuan Basin began, and the geological research and petroleum exploration in the shallow-water carbonate platform area adjacent to the trough have been paid attention [27,28]. Moreover, the research on unconventional oil and gas in the Maokou Formation has also made great progress. Recently, the industrial daily gas flow of 1.68 × 10^4^m^3^ and 3.06 × 10^4^m^3^ has been obtained individually from Well JS1 and Well YH1 in the Fuling area, southeastern Sichuan Basin [29], making the Maokou Formation another ideal series with conventional–unconventional oil and gas under the control of platform–trough evolution. So far, exploration in the Maokou Formation has shown a vigorous situation of basin wide, multi types and multi domains, and has become one of the hottest series for oil and gas exploration in the Sichuan Basin [5]. In this context, to further elaborate the platform–trough sedimentary pattern of the Maokou Formation and clarify the distribution of conventional and unconventional oil and gas resources, the regional sequence stratigraphic distribution and lithofacies paleogeographic characteristics need to be studied, and the coupling relationship between tectonic–sedimentary differentiation and reservoir formation needs to be determined. However, due to the unavailability of early drilling, coring and seismic data, as well as the continuous tectonic–sedimentary differentiation during the Maokou Formation depositional period, the sedimentary evolution process of the Maokou Formation in northeastern Sichuan Basin became very complex. There is no unified understanding of the uplift–depression pattern and sedimentary differentiation process of the Maokou Formation depositional period, and the vertical and horizontal distribution of sedimentary facies, sedimentary evolution and its petroleum geological significance remain unclear, which undoubtedly limits the exploration process of the Maokou Formation. In recent years, with the continuous enrichment of drilling and seismic data and the breakthroughs in non-traditional technologies such as identifying formation interfaces using wavelet transform and Fourier transform [30,31], it has provided the possibility to further clarify the formation distribution and lithofacies paleogeographic characteristics of the Maokou Formation.

Based on field outcrop, drilling, logging and seismic data, the sequence–lithofacies paleogeographic evolution and its control on reservoir types were discussed. The research results lay a geological foundation for future oil and gas exploration in the Maokou Formation, and provide a typical example of marine oil and gas distribution controlled by the platform–trough sedimentary geomorphic pattern formed under the effect of tectonic–sedimentary differentiation.

## Section1: Geologic setting

**Fig 1:**
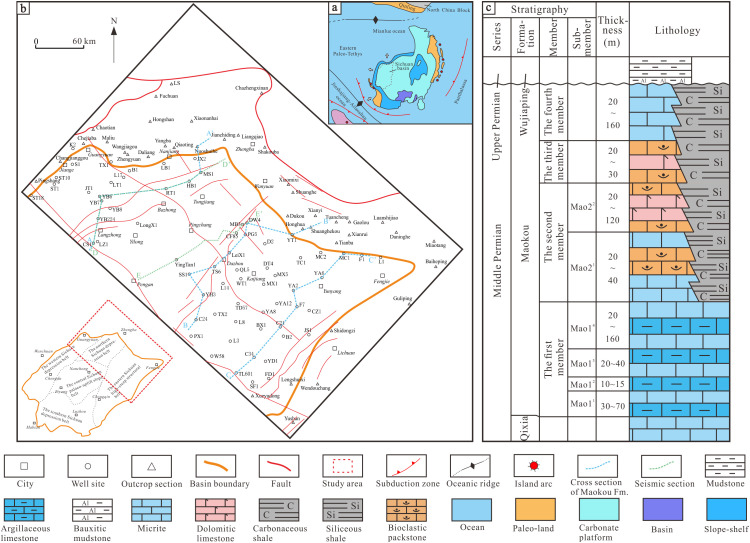
Geology overview and composite stratigrapic column of Maokou Formation in northeastern Sichuan Basin.

The Sichuan Basin in the northwest of the Yangtze Paraplatform is a rhombic sedimentary–tectonic superimposed basin formed by the northeast and northwest crossing deep-large faults in the paraplatform. It began to take shape during the Indosinian, and then formed the present tectonic features through the intense compresso-shear faulting–folding activities during the Himalayan [32–34]. During the Permian, the Sichuan Basin was bounded by the Mianlue Ocean in the north across the sea from the North China Plate, the Jinshajiang–Ailaoshan Ocean in the south/southwest across the Indosinian and Qiangtang blocks, and the vast Songpan–Ganzi Ocean Basin in the west (Fig 1a) [35,36]. The tectonic evolution of the Mianlue Ocean Basin experienced the initial opening and formation of the Devonian–Carboniferous ocean basin, and the Permian and Triassic mature ocean basin. With the initial collision of the South China Plate and North China Plate on the eastern margin of the Yangtze Block during the Late Permian, the Mianlue Ocean began to close from east to west [37,38]. The study area is located in the northeast of the Sichuan Basin, starting from Peng’an in the west, reaching Wanyuan in the east, Guangyuan in the north, and Lichuan in the south. The tectonic location includes the northern margin of the western Sichuan depression belt, the northern Sichuan depression belt, and the eastern margin of the eastern Sichuan high-steep structural belt (Fig 1b). In the Middle Permian Guadalupian, marine carbonate rocks were developed in the Upper Yangtze region, and the regional stratum was named the Maokou Formation. From bottom to top, it can be divided into four lithologic members: Mao-1, Mao-2, Mao-3 and Mao-4. Specifically, the Mao-1 Member can be subdivided into four sub-members: Mao-1^1^, Mao-1^2^, Mao-1^3^ and Mao-1^4^, and the Mao-2 Member can be subdivided into Mao-2^1^ and Mao-2^2^ sub-members (Fig 1c). In the early stage of the Middle Permian Maokou Formation, the largest marine transgression event since the Permian occurred in the Sichuan Basin [39]. A set of “eyeball–eyelid” limestone was mainly deposited in the Mao-1 Member in deeper water facies in the study area [19,40,41]. In the middle stage of the Maokou Formation sedimentation, affected by marine regression, the water energy was high. The lower Mao-2 sub-member mainly developed bioclastic limestone, with thin grained dolomite seen in some areas. In the late stage of the Maokou Formation sedimentation, with the extension and closure of the continental margin on the southern side of the Mianlue Ocean and the continuous uplift of Emei mantle plume, the geomorphic setting with several alternating uplifts and depressions were formed in the basin [15,42,43]. Typically, in northeastern Sichuan Basin, under the strong tension [44], apparent tectonic–sedimentary differentiation occurred on the east and west sides, with the deposits as carbonates such as micritic/bioclastic limestone in the upper Mao-2 to Mao-4 Members in the west of the study area, transiting gradually to the carbonaceous and siliceous muddy shales in the Gufeng Member [34,45].

## Section2: Sample and methods

The data sets used in this study include: outcrop data, well logging data, regional seismic data. The samples used for lithology description, sedimentary facies analysis, and analysis of petrophysical properties are from sixty single wells, three drilling cores, and three outcrop profiles; among which Twenty-four wells and two outcrop profiles were used for cross–well correlation, namely the southwest-northeast (SW–NE) correlation. Microscopic thin sections were made from 90 samples collected from three drilling wells and two outcrops, which were polished (30 μm thick) at the Key Laboratory of Carbonate Reservoirs, China National Petroleum Corporation, Research Branch of Southwest Petroleum University. An Upright Polarizing Microscope Leica DM4 P was utilized to observe and illustrate the thin sections for facies and petrographic descriptions. The seismic data were acquired for oil and gas exploration, the horizon and seismic facies analysis was accomplished using Landmark software. By combining seismic and logging data, a well – seismic framework profile is established to further constrain the lateral changes and plane distribution of strata in the study area

## Section3: Result

### Division of stratigraphic sequences

#### Stratigraphic sequence boundary identification.

**Fig 2:**
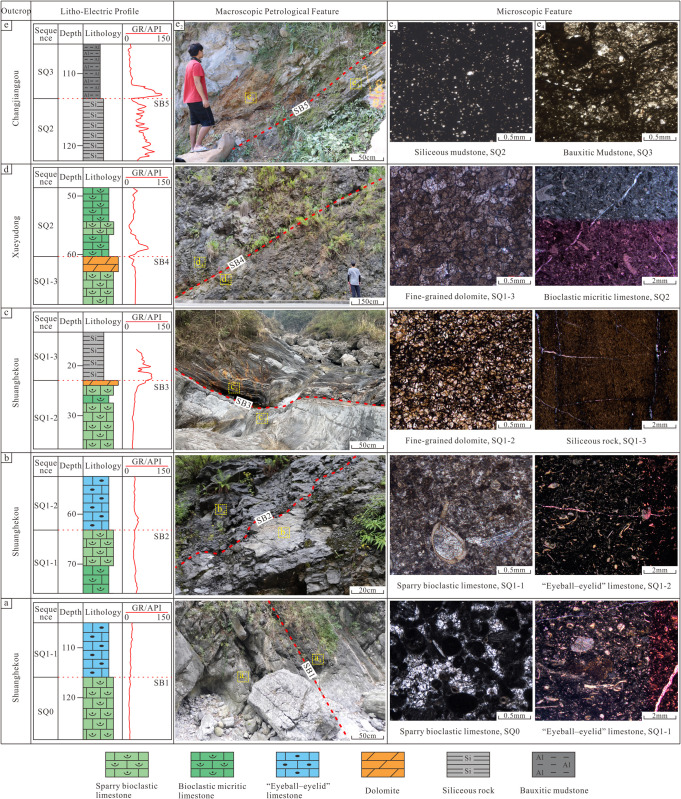
Identification signs of the sequence boundaries of the Maokou Formation in northeastern Sichuan Basin.

This study identified five sequence boundaries (SB1–SB5) within the Maokou Formation (Fig 2), based on high-resolution natural gamma-ray logging data from field outcrops and characteristic macro- to microscopic petrological features. These boundaries comprise three third-order sequence boundaries (SB1, SB4, SB5) and two fourth-order sequence boundaries (SB2, SB3). SB1 and SB5, corresponding to the bottom and top boundaries of the Maokou Formation, respectively, are the regionally exposed unconformable surfaces [46,47]. The strata below SB1 are dominated by sparry bioclastic limestone of the Qixia Formation, which frequently exhibits a leopard-spotted texture due to karst modification. Above SB1, the Maokou Formation is characterized by “eyeball-eyelid” limestone, accompanied by a slight positive deflection in the GR curve (Fig 2a). SB5 is an exposure surface at the top of the Maokou Formation. Below the boundary, siliceous mudstone is developed, while the overlying strata are typically marked by the Wangpo Shale. The GR curve exhibits a distinct positive deflection at this interface (Fig 2e).

SB2 is a typical lithofacies transition surface. A marked lithological shift is observed between the grayish-white, medium-thick bedded sparry bioclastic limestone below SB2 and the “eyeball-eyelid” limestone above SB2 (Fig 2b). Microscopic analysis further reveals significant argillaceous material and abundant well-preserved bioclasts in the overlying interval, indicative of low-energy transgressive deposition. The GR curve exhibits a sharp transition, initiating a pronounced positive deflection from its previous box-shaped low values.

Below SB3, grayish-white medium and thick layers of grainstone or dolomite were observed macroscopically (Fig 2c). Locally, vertical solution grooves formed by karstification are present. Microscopic analysis reveals argillaceous infill between dolomite crystals, attributed to mud deposition in the overlying transgressive low-energy depositional setting. Above SB3, banded siliceous mudstone dominates, containing abundant limestone collapse breccia. The GR curve displays a distinct positive deflection at this boundary.

Below SB4, thick-bedded, massive grainstone or dolomite is developed, exhibiting abundant bed-parallel dissolution vugs macroscopically, indicative of intense karst modification (Fig 2d). Microscopic observations reveal well-developed intercrystalline dissolution pores. Above SB4, dark gray bioclastic micritic limestone dominates, containing bioclasts such as echinoderms and brachiopods (Fig 2d). The GR curve shows a sharp positive deflection initiated from low baseline values, marking a transition from high-energy to low-energy depositional conditions.

#### Stratigraphic sequence division scheme.

Previous researchers have conducted extensive studies on the stratigraphic sequence division of the Middle Permian Maokou Formation in the Sichuan Basin. Based on field outcrops, core and cuttings data, well logging records, and integrated with methods such as stratigraphic sequence boundary identification and typical seismic reflection profiles, these investigations have proposed two main schemes: one dividing the Maokou Formation into two third-order sequences and the other into three third-order sequences [48–50]. The primary reason for this discrepancy lies in the fact that previous division schemes were based on the assumption that the top of the Maokou Formation in the study area had undergone significant erosion. However, this study reveals that the stratigraphic differentiation of the Maokou Formation in the northeastern Sichuan Basin is not erosion-induced, but is instead related to tectonic-sedimentary differentiation processes that drove variations in sedimentary facies.

**Fig 3:**
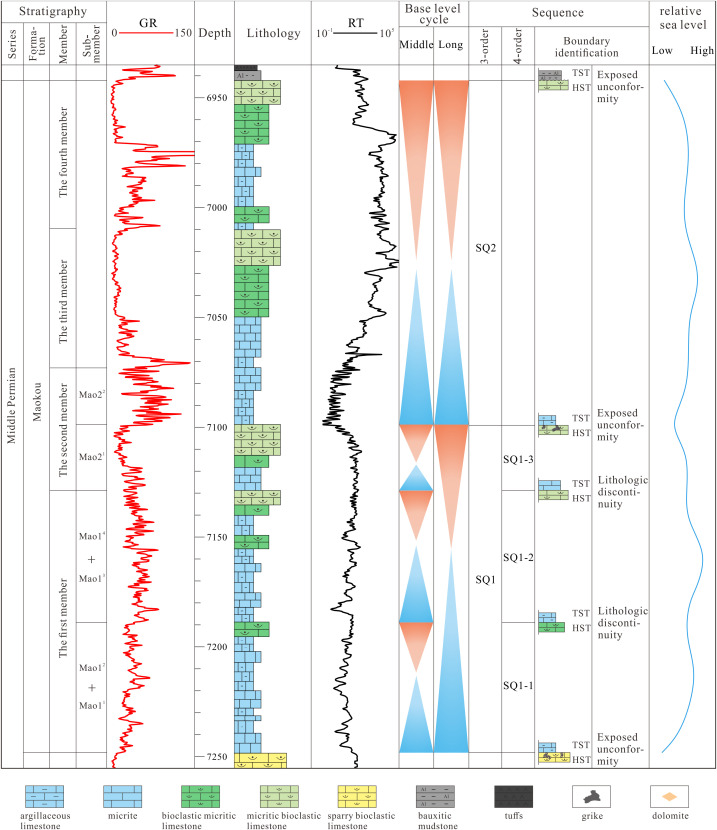
Composite stratigraphic column of sequence stratigraphy of Middle Permian Maokou Formation in Well YB7 in northeastern Sichuan Basin.

The sequence stratigraphic division of the Maokou Formation fundamentally reflects depositional responses governed by eustatic sea-level cycles. This conclusion is corroborated by the positive-negative trends and inflection points of the INPEFA_GR curve, as well as Fischer diagrammatic analysis [48,49]. Both methodologies collectively demonstrate the presence of two complete, higher-magnitude actual accommodation cycles within the Maokou Formation, thereby validating its subdivision into two third-order sequences. Based on the aforementioned identification of sequence boundaries, combined with depositional cycles and rock microfacies assemblage units, the Maokou Formation is divided into two third-order sequences (SQ1 and SQ2), and SQ1 is subdivided into three fourth-order sequences (SQ1–1, SQ1–2, and SQ1–3). Specifically, SQ1 corresponds to the Mao-1^1^–Mao-2^1^, and SQ2 corresponds to the Mao-2^2^–Mao-4; SQ1–1, SQ1–2, and SQ1–3 correspond to the Mao-1^1^ + Mao-1^2^, Mao-1^3^ + Mao-1^4^, and Mao-2^1^ sub-members, respectively (Fig 3).

### Characteristics of sequence stratigraphy

Based on the sequence division of the Permian Maokou Formation in northeastern Sichuan Basin, typical wells and sections with high-precision GR data in the study area were selected for lateral correlation of sequence stratigraphy, and a sequence stratigraphic framework was established. Combined with 2D and 3D seismic data in the study area, seismic framework sections were extracted more accurately to display the filling process and depict the planar distribution of sequence stratigraphy.

#### Sequence stratigraphic framework.

**i). Correlation framework sections of drilling–outcrop sequence stratigraphy**

In order to better study the filling process and planar distribution pattern of the Maokou Formation sequence in northeastern Sichuan Basin, three typical SW–NE trending sections in the study area were selected for correlation, and the top of the SQ1 sequence was flattened to reveal the filling characteristics of the Maokou Formation sequence.

**Fig 4:**
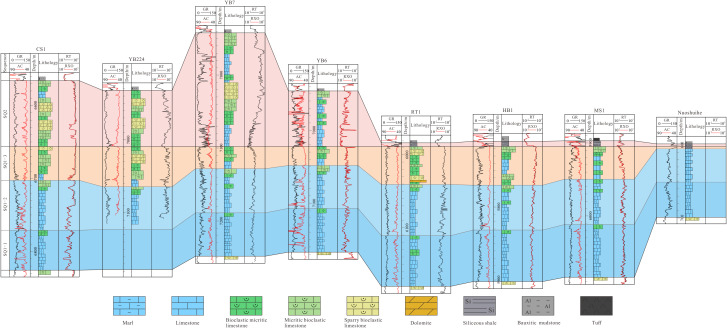
Stratigraphic sequence of Maokou Formation crossing CS1–YB224–YB7–YB6–RT1–HB1–MS1–Nuoshuihe (The section position is shown in Fig 1).

Section No.1 (A–A’) is located in the north of the study area and consists of eight sections: Wells CS1, YB224, YB7, YB6, RT1, HB1, MS1 and section Nuoshuihe. In the lateral correlation section in Fig 4, both SQ1–1 and SQ1–2 exhibits a characteristic of “thick in the middle and thin on both sides”. From southwest to northeast, the stratal thickness progressively increases before sharply decreasing. SQ1–3 exhibits a characteristic of “alternating thick and thin zones”. Thickened zones are observed in the CS1-YB224 and RT1-MS1, while thinned zones occur in the YB7-YB6. The Nuoshuihe outcrop displays extremely thin strata. SQ2 varies significantly in thickness, with an overall characteristic of “thick in the west and thin in the east”. Its thickness is the largest in YB7, the smallest in Nuoshuihe section, sharply decreases from YB6 to LT1, and continues to slightly decrease along the northeast direction.

**Fig 5:**
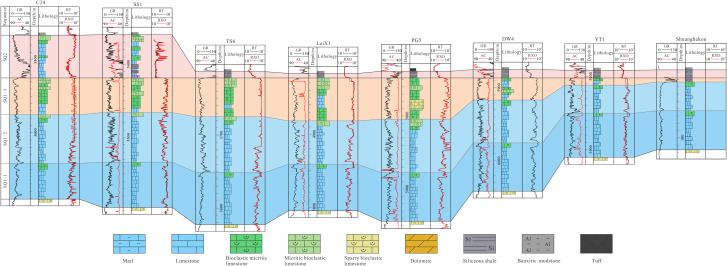
Stratigraphic sequence of Maokou Formation crossing C24–SS1–TS6–LeiX1–PG5–DW4–YT1–Shuanghekou (The section position is shown in Fig 1).

Section No.2 (B–B’) is located in the center of the study area and consists of 9 sections: Wells C24, SS1, TS6, LX1, PG5, DW4, YT1 and section Shuanghekou. In the lateral correlation section in Fig 5, both SQ1–1 and SQ1–2 exhibits a characteristic of “thick in the middle and thin on both sides”. From southwest to northeast, the strata thickness gradually thickens and then gradually thins. It is worth mentioning that the thickness of SQ1–2 decreases sharply from DW4 along the northeast direction to YT1 and Shuanghekou sections. The overall thickness of SQ1–3 has the characteristic of “slightly increasing and then rapidly decreasing” from southwest to northeast. Specifically, the overall thickness change from C24 to LeiX1 is not significant, with relatively slight alternating thickness characteristics in some areas. At PG5, SQ1–3 begins to thicken significantly and reaches its maximum value. From the PG5 well area along the northeastward direction to the DW4 well area, the thickness decreased sharply and continued to develop to the Yingtan 1 well and the Shuanghekou section, with the thickness thinning to 10 meters. The thickness of SQ2 still has the characteristics of “thick in the west and thin in the east”, but compared with Section No.1, the thickness on the west side is thin, while the thickness difference on the east side is not significant. The thickness of SQ2 from C24 to SS1 is large and stable, while from SS1 to TS6, it begins to rapidly thin and continues to develop along the northeast direction to the Shuanghekou section.

**Fig 6:**
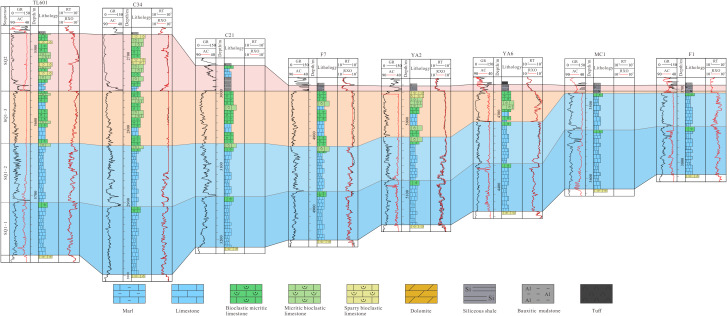
Stratigraphic sequence of Maokou Formation crossing TL601–C34–C21–F7–YA2-YA6–YA19–MC1–F1 (The section position is shown in Fig 1).

Section No.3 (C–C’) is located in the south of the study area and consists of nine sections: Wells TL601, C34, C21, F7, YA2, YA6, YA19, MC1 and F1. In the lateral correlation section in Fig 6, SQ1–1 exhibits a characteristic of “thick at both ends and thin in the middle”, with the thick value area mainly in TL601, C34 and MC1, and the small-thickness value area mainly from F7 to YA19. SQ1–2 has the characteristics of “thick in the southwest and thin in the northeast”, that is, from TL601 to F1 along the northeast direction, SQ1–2 tends to thin gradually. The overall thickness variation in SQ1–3 inherits from SQ1–2, thinning from southwest to northeast. Unlike SQ1–2, the thickness of SQ1–3 in TL601 to YA2 does not show significant changes, with only a slight thinning trend. From YA2 to the northeast, it begins to show a sharp thinning trend and continues to develop to F1. Similar to Section No.2, this section reveals that the thickness of SQ2 also has the characteristic of “ thick in the west and thin in the east “, among which there is a significant thinning from C21 to F7.

**ii). Seismic sequence correlation framework sections**

**Fig 7:**
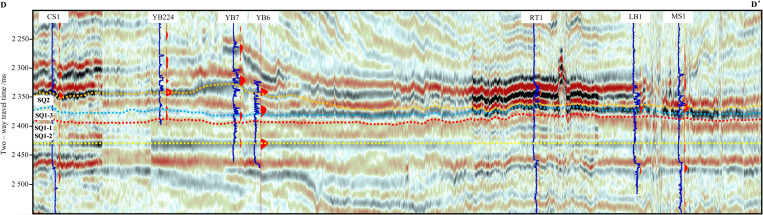
Seismic section through Wells LZ1–LX1–RT1–HB1–MS1 in the north of the study area (Pre-stack time offset data. The section position is shown in Fig 1).

**Fig 8:**
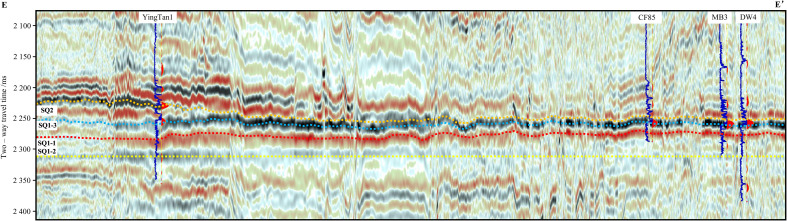
Seismic section through Wells YingTan1–CF85-MB3-DW4 in the middle of the study area (Pre-stack time offset data. The section position is shown in Fig 1).

In order to accurately describe the sequence stratigraphic characteristics of the Maokou Formation in northeastern Sichuan Basin, two SW–NE 3D seismic sections (D–D ‘and E–E’) were selected in the north and middle of the study area. For the seismic section in the north of the study area, we incorporated drilling data from Wells CS1, YB224, YB7, YB6, RT1, HB1 and MS1 were used. For the seismic section in the middle, we incorporated drilling data from Wells YingTan1, CF85, MB3 and DW4 were used. Synthetic seismograms (well-to-seismic calibration) were generated using acoustic travel-time differences and bulk density data from these wells, enabling precise identification of the seismic reflection characteristics of the Maokou Formation’s top and basal boundaries and facilitating the tracing of stratigraphic horizons. Meanwhile, to better visualize lateral variations in the total thickness of the Maokou Formation, both seismic section were flattened on the base of the Maokou Formation (Figs 7 and 8). The results show that the sequence stratigraphic thicknesses of SQ1-1 and SQ1-2 are relatively stable. Both seismic profiles exhibit an overall “southwest-thin and northeast-thick” pattern, with localized minor alternating thick-thin variations. SQ1-3 is generally characterized by “thick in southwest and thin in northeast”. Specifically, section D–D’ shows that the sequence stratigraphy on the west side of YB224 is thicker, and locally has the seismic reflection characteristics of blank, disordered and poor continuity. The formations to the northeast direction of YB224 are thinned and then thickened, and the formations in RT1 are thickened obviously, and then thinned continuously along the northeast direction (Fig 7). Section E–E’ shows the thickness variation similar to section No.1, and the large-thickness value areas are in the southwest direction of YingTan1 and CF85 respectively (Fig 8). During SQ2 depositional period, the two seismic sections showed thickened platform, and the formation thickness in the northeast direction decreased rapidly. The thickness conversion zone was located in the northeast direction of YB6 and YingTan1. In terms of seismic reflection characteristics, the zones above the platform have continuous reflection characteristics with medium–strong amplitude, while the zones below the platform show the characteristics of strong amplitude, high continuity and low frequency. In general, from the thickness changes reflected by the seismic framework sections, the east and west sides of the study area had been characterized by tectonic–sedimentary differentiation during the middle–late sedimentation period of the Maokou Formation. To the sedimentary end of the Maokou Formation, the difference in stratum thickness was more obvious, and the sedimentary differentiation between the east and west sides was intensified.

#### Plane distribution of sequence stratigraphy.

**Fig 9:**
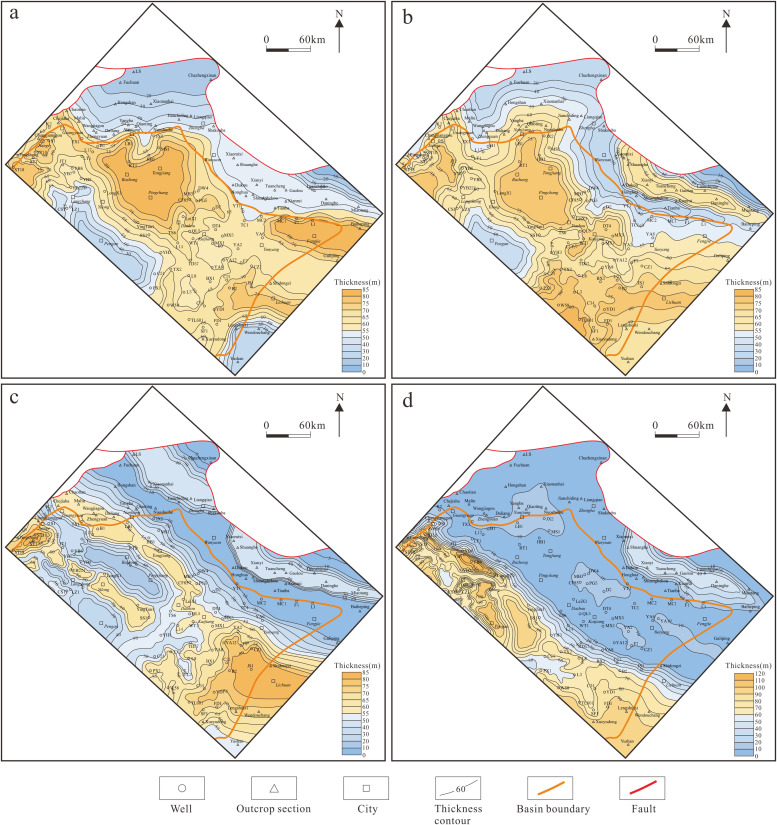
Thickness maps of sequences of Maokou Formation in northeastern Sichuan Basin.

Based on the above drilling-outcrop and seismic sequence correlation framework sections, the sequence stratigraphic thickness data of each sequence of the Maokou Formation in wells and outcrops in the study area were further sorted out (S1 Table in S1 File), and the thickness maps of SQ1–1, SQ1–2, SQ1–3 and SQ2 were compiled (Fig 9) to show the sequence stratigraphic filling process and plane distribution characteristics.

SQ1–1 is 0–85 m thick, with the characteristics of “thick in the middle and thin on both sides” (Fig 9a). The large-thickness value areas are mainly distributed in the Jiange–Bazhong–Kaijiang–Lichuan and Fengjie and its periphery, with thickness generally greater than 70 m. To the west, in the Langzhong-Pengan area, the thickness is 30–55 m; to the east, in the Zhenba-Wanyuan area, the thickness is generally less than 60 m, and less than 10 m locally. The thickness difference between the east and west sides of the study area during SQ1–2 depositional period occurred initially.

SQ1–2 is 0–85 m thick, with the plane distribution inherited from SQ1–1, that is, being thick in the middle and thin on both sides (Fig 9b). Its thickness is relatively large in the Bazhong–Pingchang–Kaijiang–Lichuan area (generally >65 m). To the west, the thickness gradually decreases (generally <55 m); to the east, the thickness decreases, then increases, and finally sharply decreases, with the thickest part being 65 m and the thinnest part being less than 10 m. The thickness difference between the east and west sides was gradually significant during SQ1–2 depositional period.

SQ1–3 is 0–80 m thick, showing a distribution pattern of “alternating thick and thin zones” from southwest to northeast (Fig 9c). Its thickness is large in areas of Jiange-Cangxi-Yilong, Guangyuan-Tongjiang-Kaijiang, Xiaomixi-Gaolou-Miaotang, Lichuan and Zhenba (generally >55 m), and small in areas of Langzhong-Pengan, Bazhong-Pingchang, Nuoshuihe-Wanyuan-Fengjie and western Zhenba (generally <30 m). The thickness difference between the east and west sides became further larger during SQ1–3 depositional period.

SQ2 is 0–135 m thick, with the general characteristic of “thick in the west and thin in the east”. The thickness difference between the east and west sides of the study area reaches the peak (Fig 9d). Specifically, to the west of Wells JT1–LX1–YingTan1–QL42–JS1, the formation thickness is generally greater than 50 m, and the local thickness varies greatly. For example, its thickness in Well YB7 can reach 130 m. The thickness to the east of the area is sharply reduced to less than 20 m, and only the thickness in some areas such as Shuanghe–Gaolou–Daninghe is more than 30 m.

Generally, the sequence stratigraphy in the study area during the SQ1–1 and SQ1–2 depositional periods was “thick in the middle and thin on both sides”, and the thickness difference between the east and west sides occurred initially. The thicknesses during the SQ1–3 depositional period had the characteristics of “alternating thick and thin zones”, and the thickness difference between the east and west sides was gradually significant. During the SQ2 depositional period, the sequence stratigraphy was “thick in the west and thin in the east”, and the thickness difference between the east and west sides reached the peak. From the initial to the final stages of Maokou Formation deposition, the thin-thickness zones of the strata continuously extended basinward, while the southwest-northeast thickness differentiation became increasingly pronounced.

### Lithofacies paleogeographic characteristics in sequence framework

#### Types and characteristics of sedimentary facies.

Previous studies have shown that the Maokou Formation is mainly composed of limestone, marl, dolomite, siliceous limestone, siliceous rock and muddy shale, and is rich in gastropods, brachiopods, algae and bivalve fossils. According to the identified types and characteristics of carbonate rocks, and with reference to the lithofacies, paleogeography and sedimentary model of the Permian Maokou Formation in the Sichuan Basin [51,52], it is considered that the sedimentary system of the Middle Permian Maokou Formation in northeastern Sichuan Basin can be divided into two types of carbonate platform models: carbonate ramp and rimmed carbonate platform. The carbonate ramp was mainly developed during the SQ1–1 and SQ1–2 depositional periods. After the formation of deep-water platform-basin due to the intensification of tectonic–sedimentary differentiation during the SQ1–3 depositional period, the sedimentary system was transformed into rimmed carbonate platform. For detailed and comprehensive analysis of sedimentary facies, the sedimentary facies in the study area are divided into semi-restricted platform, platform margin, slope, platform-basin and ramp (Table 1).

**Table 1:** Types and depositional periods of sedimentary facies in northeastern Sichuan Basin.

Platform type	Facies	Subfacies	Microfacies	Depositional period
Rimmed carbonate platform	Semi-restricted platform	Intra-platform shoal	High-energy and low-energy bioclastic shoals	SQ1–3, SQ2
		Semi-restricted sea	Limy semi-restricted sea	
		Intra-platform depression	Marly intra-platform depression	
	Platform margin	Platform margin shoal	High-energy and low-energy bioclastic shoals	
		Inter-shoal sea	Limy inter-shoal sea	
	Slope			
	Platform-basin			
Carbonate ramp	Ramp	Middle ramp		SQ1–1, SQ1–2
		Outer ramp

**i). Ramp**

**Fig 10:**
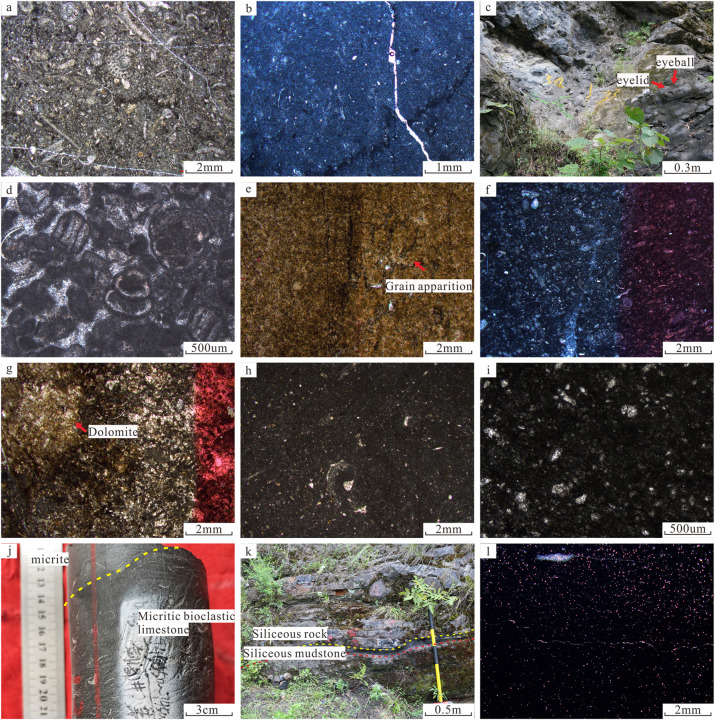
Macroscopic and microscopic photos of sedimentary microfacies of Maokou Formation in northeastern Sichuan Basin. (a) Packstones, plane-polarized light, Shuanghekou section, SQ1-1; (b) Micritic limestone, plane-polarized light, Well YingTan1, 6477.8 m, SQ1-2; (c) “Eyeball–eyelid” limestone, Zhengyuan section, SQ1-1; (d) Sparry bioclastic limestone, plane-polarized light, Zhengyuan section, SQ1-3; (e) Fine-grained dolomite, plane-polarized light, with particle phantom, Well S1, 5286.2 m, SQ1-3; (f) Bioclastic micritic limestone, plane-polarized light,Well YingTan1, 6478.35 m, SQ1-3; (g) Spotted dolomitic limestone, plane-polarized light, dyed by sodium alizarinsulfonate, Well B1, 5906.52 m, SQ1-3; (h) Bioclastic-bearing micritic limestone, plane-polarized light, Well S1, 5299.23 m, SQ1-3; (i) Micritic limestone, plane-polarized light, Lengshuixi section, SQ1-3; (j) Micritic bioclastic limestone in the lower part and micritic limestone in the upper part, being storm sediment, Well JG1, 7450.5 m, SQ2; (k) Interbedded siliceous rock and siliceous mudstone, Zhengyuan section, SQ2; (l)Siliceous mudstone, plane-polarized light, Shuanghekou section, SQ2.

The ramp facies is only observed in SQ1–1 and SQ1–2. Carbonate ramp is predominant in the main part of the study [53], which can be subdivided into two subfacies: middle ramp and outer ramp. Between the normal wave base and the storm wave base is the middle ramp, with relatively strong hydrodynamic conditions. There are various fossil organisms, and the lithology is mainly composed of packstones, micritic limestone, and argillaceous limestone(Fig 10a). The outer ramp, below the storm wave base, is less affected by storm action, and mainly composed of deep-water low-energy carbonate rocks such as argillaceous limestone and micritic limestone, with rare bioclasts (Fig 10b). Micrites with a very small content of organisms mainly formed in a low-energy lentic environment, such as the outer ramp below a storm wave base. Owing to extensive transgression at the early depositional stage of the Maokou Formation, thick marls were deposited at the bottom of the Maokou Formation (Fig 10c).

**ii). Platform margin**

The platform margin facies includes two subfacies: platform margin shoal and inter-shoal sea. The platform margin shoal, developed during the SQ1–3 and SQ2 depositional periods, is mainly composed of thick, massive, light-gray grainstone, and presents box-shaped GR logs, with relatively low GR value. As observed under the microscope, this lithofacies has particle content greater than 50%, with the dominance of bioclastic particles, including brachiopods, echinoderms, and large fusulinid fossil fragments, with sparry cements between particles (Fig 10d). In addition, the typical difference from other sedimentary facies zones is that the grainstones at the platform margin shoal are universally dolomitized, with the common occurrence of fine–medium crystalline dolomite (Fig 10e). Inter-shoal sea is distributed in relatively low-energy areas between the platform margin shoals, and mainly composed of micritic limestone and bioclastic micritic limestone (Fig 10f).

**iii). Semi-restricted platform**

The semi-restricted platform facies includes three subfacies: intra-platform shoal, semi-restricted sea, and intra-platform depression. The intra-platform shoal is located in the underwater uplifted area with positive topography from the normal wave base to the average low tide line inside the platform, with major lithology of sparry bioclastic limestone and micritic bioclastic limestone. The intra-platform shoals were significantly influenced by early diagenetic exposure karstification and dolomitization, resulting in a high frequency of mottled dolomitic limestone development (Fig 10g). According to the differences in rock types, the intra-platform shoal can be subdivided into low-energy intra-platform shoal and high-energy intra-platform shoal. The former is mainly composed of micritic bioclastic limestone, while the latter is mainly composed of sparry bioclastic limestone, both types have been variably modified by karstification and dolomitization. The semi-restricted sea is widely distributed in carbonate platform, mainly in a still water low-energy environment below the wave base and sandwiched between grain shoals within the platform. It is generally below the average wave base and mainly composed of bioclastic-bearing micritic limestone and micritic limestone (Fig 10h). The intra-platform depression is a relatively low-lying area between the shoals within the platform, in a low-energy environment below the average wave base, and mainly composed of marl and micritic limestone (Fig 10i).

****iv).** Slope**

The slope facies is only developed in SQ1–3 and SQ2, generally between the normal wave base and the average storm wave base, having platform-basin sediments with low energy in deep waters on one side and shallow-water carbonate platform sediments with high energy in shallow-waters on the other side. This environment usually has the characteristics of weak water energy, low wave action, and relatively stable water, with major lithology of dark gray thin-to-medium-bedded bioclastic-bearing argillaceous limestone. It hosts diverse and abundant fossil assemblages, including small planktonic foraminifera, brachiopods, and gastropods. In addition, if affected by storm action, the slope facies may develop low-energy grain shoals dominated by micritic bioclastic limestone (Fig 10j).

****V).** Platform-basin**

The platform-basin facies is only revealed in SQ1–3 and SQ2. It records an extremely low-energy environment, with water depths of tens or even hundreds of meters, below the storm wave base. In the study area, the platform-basin facies of the Maokou Formation is characterized by thin-bedded black siliceous rocks, siliceous mudstone or calcareous mudstone. Macroscopically, uneven interbedding of siliceous rocks and siliceous mudstone is observed (Fig 10k). Microscopic analysis reveals typical deep-water siliceous radiolarians and sponge spicules. Additionally, thin-bedded calcareous mudstone from the Shuanghekou section exhibits well-developed horizontal bedding, with abundant calcareous silt-sized particles visible under the microscope (Fig 10l).

#### Vertical and horizontal distribution of sedimentary facies.

**Fig 11:**
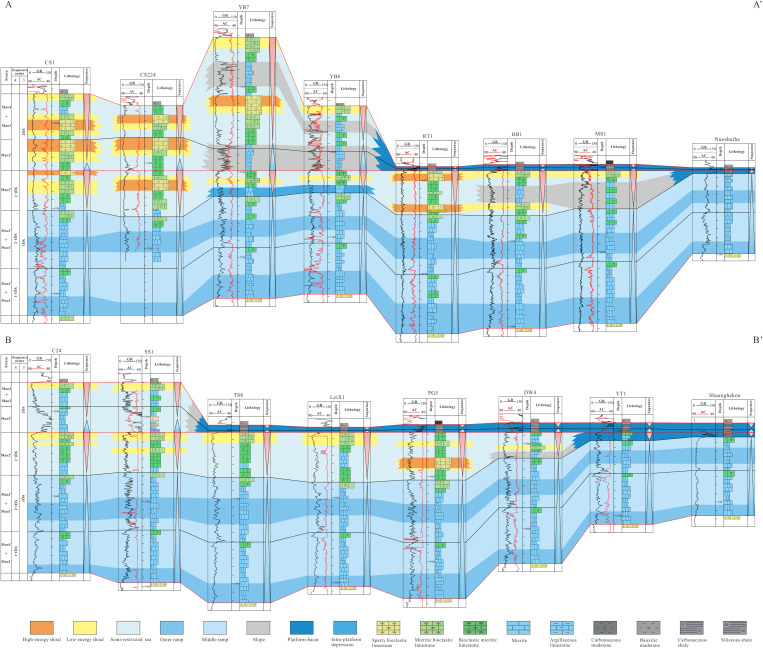
Cross-well sedimentary facies sections of Maokou Formation in northeastern Sichuan Basin (The section position is shown in Fig 1).

The SW–NE sedimentary facies section of the Maokou Formation (Fig 11) shows that, during the SQ1–1 and SQ1–2 depositional periods, ramp facies was dominantly developed in the study area, with major lithology of micritic mudstone and argillaceous limestone, often occurring in a rhythmical interbedded pattern of limestone and marl. The grain shoal was generally underdeveloped in these deposition periods, but only horizontally stable thin low-energy shoals with major lithology of micritic bioclastic limestone was developed in the late stage of SQ1–2 depositional period. Based on the characteristics of lithology and lithologic associations, the ramp facies can be subdivided into outer ramp and middle ramp subfacies. The outer ramp is mainly composed of argillaceous limestone, while the middle ramp is mainly composed of micritic limestone, occasionally with argillaceous limestone and bioclastic micritic limestone. During the SQ1–3 depositional period, the study area mainly developed high-energy shoal, low-energy shoal, semi-restricted sea, intra-platform depression, slope, and platform-basin. Specifically, the high-energy shoal is revealed in the CS1 and YB224 well blocks in the western part of the study area, and in the RT1 and PG5 well blocks in the central part of the study area, and it is lithologically composed of sparry bioclastic limestone. The low-energy shoal is generally distributed in a consistent pattern with the high-energy shoal, but it is more widespread, with poor lateral continuity and varying thickness. It is relatively thick in the HB1 and LeiX1 well blocks, and lithologically composed of micritic bioclastic limestone. During the SQ2 depositional period, the western part of the study area mainly developed high-energy shoal, low-energy shoal, and semi-restricted sea sediments. Specifically, high/low-energy shoals are mainly developed in the CS1, YB224, and YB7 well blocks, and lithologically composed of micritic bioclastic limestone and sparry bioclastic limestone. Affected by the NW–SE faulting activity and tectonic–sedimentary differentiation, the eastern part (to the east of Wells YB6 and SS1) of the study area is mainly composed of platform-basin sediments, with siliceous shale and siliceous mudstone as the main lithologies.

Overall, both of the above two sedimentary facies sections show that the sedimentary periods of SQ1–1 and SQ1–2 were stable gentle slope facies deposits, and the grain shoal was underdeveloped. The SQ1–3 depositional period was marked by tectonic subsidence, leading to the evolution of the eastern study area into a deep-water platform basin depositional environment. Grain shoals were restricted to the central and western regions during this phase. By the SQ2 depositional period, the deep-water platform basin facies further expanded from the northeast to the southwest, while the extent of shallow-water platform deposition continued to diminish. Compared to the SQ1–3 period, the grain shoals exhibited an overall westward migration trend.

#### Lithofacies paleogeographic features of sequences.

**Fig 12:**
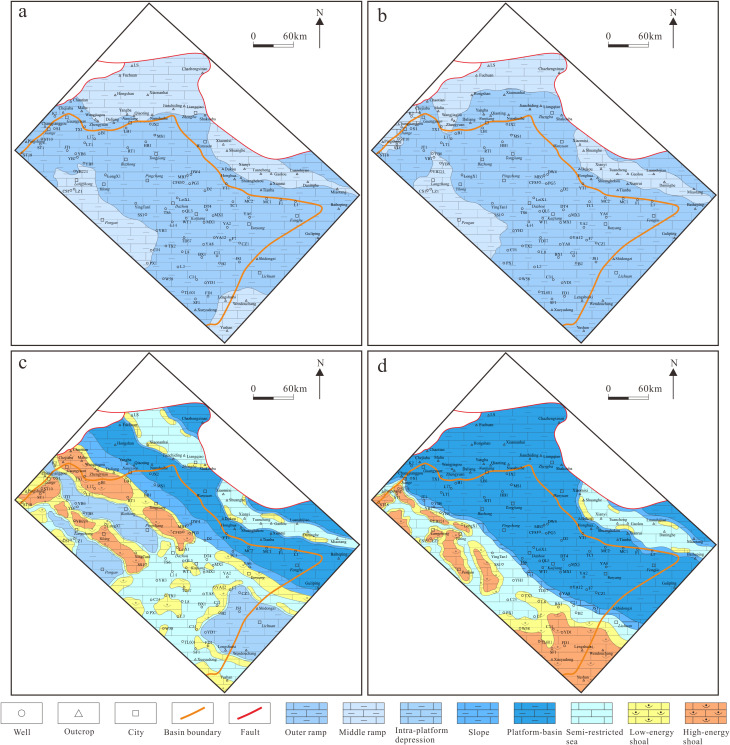
Lithofacies paleogeographic characteristics of sequences in Maokou Formation in northeastern Sichuan Basin.

Based on the sedimentary setting of the South China region and the sedimentary characteristics of the Mao-1 Member (SQ1–1, SQ1–2) in the Sichuan Basin without platform-margin reef-shoal zones, previous studies classified the Mao-1 Member in the Sichuan Basin as a carbonate ramp sedimentary model [52]. As mentioned earlier, the thickness variation of SQ1–1 and SQ1–2 sequences in the northeastern Sichuan Basin is generally small, reflecting a relatively flat sedimentary landform. The thickness gradually increases and then decreases from west to east, and the large-thickness value areas are distributed in a wide range, with relatively high particle content. This indicates that during the SQ1–1 and SQ1–2 depositional periods, outer ramp dominated the study area, mainly distributing in a NW–SE direction in the central part of the study area, while middle ramp was mainly distributed in the small-thickness value areas of the strata in the east and west of the study area (Fig 12a, 12b). Essentially, the water kinetic energy of the middle ramp is relatively high, which often redistributes the materials deposited in the middle ramp to the inner and outer ramps, and the top part of the outer ramp is the unloading zone for sediments, resulting in the thickest strata [54]. The sustained marine transgression during the early Maokou Formation facilitated the widespread development of carbonate ramp deposits across the study area during the depositional periods of SQ1–1 and SQ1–2. These intervals exhibited similar planar distribution of sedimentary facies zones, dominated by outer ramp deposits, with subordinate mid-ramp facies.

During the SQ1–3 depositional period, obvious sedimentary differentiation had occurred in the east and west of the study area in this period (Fig 12c). Deep-water sediments are developed to the east and shallow-water sediments to the west of the Nanjiang–Tongjiang–Yunyang area. Coupling with the types and characteristics of main sedimentary facies of this period mentioned above, it is believed that during the SQ1–3 depositional period, the study area developed sedimentary facies zones of semi-restricted sea, intra-platform depression, high/low-energy shoal, slope, and platform-basin, from west to east. Specifically, multiple high/low-energy shoal zones were developed in the NW–SE direction from southwest to northeast. It is worth mentioning that macroscopic and microscopic observations of the Shuanghekou section reveal the presence of siliceous nodular limestone in the early stage of SQ1–3 deposition (Fig 10k), which is a product of slump, indicating the possible existence of isolated platforms in the eastern part of the region. In addition, the SQ1–3 sequence stratigraphy in the Lichuan area in the southern part of the study area and surrounding areas has a relatively large thickness. However, analysis on the electrical properties and logging data of existing wells and the lithology and lithofacies of outcrops in the large-thickness value areas has shown that SQ1–3 reflects high GR values and is mainly composed of bioclastic micritic limestone and micritic limestone, with occasional occurrence of micritic bioclastic limestone, indicating underdeveloped grain shoal. The median-thickness value area shows more developed grain shoals. Probably, due to the continuous changes in sea level, a decrease in accommodation space induced lateral migration and growth of the shoals. As a result, a local low-energy environment was possibly formed at the tops of the shoals on the inward side due to the influence of micro-uplift barriers, resulting in a more development of granular rocks in the median-thickness value area than the large-thickness value area [55].

During the SQ2 depositional period, the formation thickness difference between the east and the west of the study area was further enlarged, and the differentiation of sedimentary facies zones became more significant, with the high-energy facies zones as a whole migrating westward (Fig 12d). Specifically, semi-restricted sea, low-energy shoal, and high-energy shoal sediments were dominant in the western part of the study area, and presented an overall NW–SW distribution. Eastward, the sediments gradually transited to slope facies. In the regions to the east of the Jiange–Yilong–Lichuan area, platform-basin became predominant, and low-energy shoal sediments appeared locally due to the inherited development of isolated platforms.

Overall, under the paleogeomorphology that was higher in the west than in the east before the deposition of the Maokou Formation, the northeastern Sichuan Basin mainly received the carbonate ramp sediments, with underdeveloped grain shoals, during the SQ1–1 and SQ1–2 depositional periods. There was significant tectonic–sedimentary differentiation in the east and west of this area during the SQ1–3 depositional period, with multiple zones of high/low-energy shoals in a NW–SE direction. The tectonic–sedimentary differentiation was further intensified during the SQ2 depositional period, associated with the westward-migrating shoals as a whole.

## Section4: Discussion

### Tectonic–sedimentary evolution

**Fig 13:**
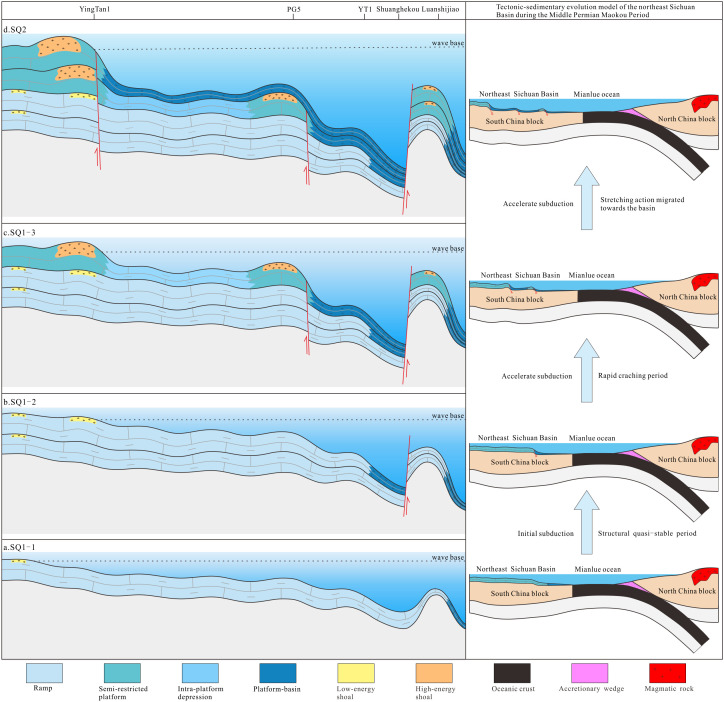
Tectonic-sedimentary evolution model in northeastern Sichuan Basin during Maokou Formation depositional period.

Through systematic researches on the filling pattern and lithofacies paleogeography of the Maokou Formation in northeastern Sichuan Basin, sustained tectonic activity and sea level eustacy are clarified as the main factors controlling the evolution of sedimentary facies zones in the Maokou Formation in northeastern Sichuan Basin. During the SQ1–1 to SQ1–2 depositional period, the Sichuan Basin was flooded by seawater due to rapid global transgression, leading to carbonate ramp sedimentation across the study area. During the SQ1–3 depositional period, with the continuous subduction and sea level decline of the Mianlue Ocean, a significant SW-NE sedimentary differentiation took place in the study area. The shoals gradually migrated southwestward, with an increase in continuous area. In the eastern part, the waters were deepened as a result of continuous N-E tensional stress and faulting activity, with deep-water low-energy sediments deposited. During the SQ2 depositional period, the strong N-E stress generated by the further subduction of the Mianlue Ocean led to an overall subsidence in the eastern part. Coupling with the rapid rise of sea level in the early stage and the slow decline of seal level in the middle and late stages, the sedimentary environment transformed into a rimmed carbonate platform, with the development of platform–platform margin–slope–platform-basin in sequence, and the shoals migrating further southwestward (Fig 13).

In the early stage of the Maokou Formation deposition, the largest regional marine flooding event occurred in South China since the Permian and even the Late Paleozoic. In the Sichuan Basin, the transgression initiated from the southeast, that is, the transgression took place northwestward from northern Guizhou and western Hubei, then from the Qinling Trench through northern Sichuan, and finally from west to east through the Longmen Mountain ancient island chain [51,56]. In this context, the study area was flooded by seawater in the early stage of the Maokou Formation deposition (SQ1−1), dominantly with filling and leveling deposition, which led to carbonate ramp sediments. Lithologically, “eyelid–eyeball” limestones were extensive, while only laminated micritic bioclastic limestones existed locally (Fig 13a). In the same period, in addition to the paleogeomorphologic inheritance from the late Qixia Formation, the tectonic–sedimentary differentiation began to take shape under the N-E extensional stress generated by the initial subduction of the Mianlue Ocean at the northern margin of the Upper Yangtze Plate. Deep-water sediments dominated by platform-basin facies began to appear locally in the east of the study area.

During the SQ1–2 depositional period, the study area was still characterized by filling and leveling deposition. Ramp deposits were inherited. On the other hand, the dominance of N-E tensional stress in the study area due to the continuous subduction of the Mianlue Ocean at the northern margin of the Upper Yangtze Plate was strengthening. As a result, the sedimentary differentiation between the east and the west was gradually intensified. To be specific, in the depression area characterized by deep water and subcompensated sedimentation, the sediments were small in thickness and mainly deep-water sediments dominated by platform-basin facies, and extended towards the basin interior to the Wanyuan area (Fig 13b). Additionally, in the Luanshijiao and its surrounding areas, the setting of underwater landform highland, coupled with the continuous influence of synsedimentary faults, allowed these areas to remain in relatively shallow-water sedimentary environments against the backdrop of the overall subsidence in the eastern part of the study area.

During the SQ1–3 depositional period, the N-E extension was further strengthened along with the continuous subduction of the Mianlue Ocean, the N-E sedimentary differentiation in the study area became more prominent, and the deep-water sedimentary area continued to expand into the basin to the Nanjiang–Tongjiang–Yunyang area (Fig 13c). The sustained and gradual N-E tensional stress resulted in a geomorphic feature with “eastward-dipping generally and graben-horst alternation locally” in the study area. In addition, the slow decline of sea level in this period induced the distribution of shoals in rows and zones in the study area. To be specific, high/low-energy shoal zones in NW-SE distribution were developed in the YingTan1 and PG5 well blocks, and the Luanshijiao area, respectively.

During the SQ2 depositional period, along with the overall expansion of the Mianlue Ocean, the N-E extensional stress reached its peak, and the subsidence range further expanded into the basin to the Jiange–Dazhou area (Fig 13d). Typically, black siliceous radiolarian argillaceous limestone, mudstone, and siliceous rocks representing deep-water platform-basin appeared in the Guangyuan–Pingchang–Kaijiang area and the Shuanghekou section [54], while Well JG1 encountered bioclastic micritic limestone and argillaceous limestone with the characteristics of storm sediments representing slope facies [57], all confirming the widespread distribution of deep-water facies in this period. The platform on the western flank of this slope facies zone also developed grain shoal sediments that extended in nearly NW–SE direction.

In summary, the synergy between the continuous expansion of the Mianlue Ocean at the northern margin of the Upper Yangtze Plate and the sea level eustacy controlled the evolution of the sedimentary facies zones in northeastern Sichuan Basin during the Maokou Formation depositional period. Especially, the N-E tensional stress generated by the expansion of the Mianlue Ocean was the main contributor to the tectonic-sedimentary differentiation in northeastern Sichuan Basin.

### Convention-unconventional gas exploration potential

As mentioned above, the study area underwent significant tectonic–sedimentary differentiation during the depositional period of the Maokou Formation due to the continuous activity of the Mianlue Ocean and the frequent sea level fluctuations. Specifically, the sedimentary differentiation was gradually intensified, triggering the gradual evolution from carbonate ramp in the early stage to alternating distribution of uplift and sag in the middle stage and finally to a platform–platform-basin sedimentary pattern. The organic-rich marls and muddy shales in the platform-basin are potential source rocks and unconventional oil and gas exploration targets. In the platform area adjacent to the platform-basin, large-scale high-energy shoals are developed, providing a material basis for conventional oil and gas exploration. In addition, influenced by diagenesis such as shoal migration, exposure, and dolomitization, high-quality high-energy shoal reservoirs can be available. Therefore, the Maokou Formation in the study area has both conventional and unconventional exploration potential.

In terms of conventional oil and gas exploration, high-energy shoal deposits are developed in both SQ1–3 and SQ2. They are distributed in a NW–SE direction, with major lithology of sparry bioclastic limestone and granular dolomite. The high-energy shoals of SQ1–3 are mainly distributed along the Jiange–Yilong and Guangyuan–Dazhou areas, while those of SQ2 mainly along the Jiange–Yilong–Lichuan area to the east. The widespread high-energy shoals provide an important material basis for the development of high-quality conventional reservoirs in the study area. Moreover, due to the high landforms where high-energy shoals were deposited, these sediments are easily exposed to form karst channels and caves during high-frequency cycles, providing dominant channels for magnesium-rich dolomitized fluids during the sedimentary diagenesis period [58], which is conducive to the occurrence of dolomitization. As dolomite is more compressive than limestone, more pores can be preserved in dolomite during the later burial process. The successful gas discoveries from high-energy shoal facies reservoirs in the western Well YT1 (SQ1–3) and the southern Wells TL6 and W67 (SQ2), with daily production rates of 7.8 × 10^4^m^3^/d, 39.8 × 10^4^m^3^/d, and 31.2 × 10^4^m^3^/d respectively, unequivocally demonstrate the conventional hydrocarbon exploration potential of the Maokou Formation. In summary, the high-energy facies zones in the study area are important for reservoir development. Additionally, the high-quality source rocks in the trough, which were developed in the same period but differ in facies from the high-energy facies zones, form an assemblage of source–reservoir in lateral contact. Thus, these facies zones stay as a good place for hydrocarbon storage, and they are expected to be large-scale exploration areas.

In terms of unconventional oil and gas exploration, several exploration wells in the Sichuan Basin have recently obtained industrial gas flows from the Mao-1 Member, with a maximum productivity of 30.01 × 10^4^ m^3^/d. The reservoir rock is mainly marl, and the storage space is mainly composed of micropores, intercrystalline pores, interlayer fractures, and microfractures, with organic pores, showing good potential for unconventional oil and gas exploration. SQ1–1 and SQ1–2 (Mao-1 Member), deposited in carbonate ramp, are thinner in the west than in the east. They are mainly composed of rhythmically interbedded limestone and marl, potentially as exploration targets for unconventional carbonate reservoirs. In addition, the tectonic–sedimentary differentiation between the east and the west was significantly intensified since the SQ1–3 depositional period, and peaked in the middle and late stages of SQ2. The most remarkable feature is the development of black organic-rich marl and muddy shale to the east of the Jiange–Yilong–Lichuan area during the SQ2 depositional period. These black organic-rich marl and muddy shale are widespread and thick to a certain extent (about 10–40 m on average), serving as a set of good source rocks. Their hydrocarbon generation potentials and organic matter enrichment patterns have been discussed in detail [29,34], indicating that this set of black rocks was formed in a shallow to semi-deep sea reduction environment with high salinity, and that they meet the criteria of source rocks (TOC > 0.50%). Moreover, drilling results also reveal a set of black organic-rich shale developed in the late stage of the Maokou Formation deposition. For instance, Well JT1 encountered 24m black siliceous organic-rich argillaceous limestones, with an average TOC content of 7.8%. In summary, the Maokou Formation in the study area has the potential for unconventional oil and gas exploration. Especially, the black rocks in SQ2 to the east of the Jiange–Yilong–Lichuan area are considered as the most favorable potential targets for unconventional oil and gas exploration.

## Section5: Conclusions

(1) The Middle Permian Maokou Formation in northeastern Sichuan Basin develops two third-order sequences (SQ1, SQ2). SQ1 can be divided into three fourth-order sequences (SQ1–1, SQ1–2, and SQ1–3). SQ1–1 and SQ1–2 are generally carbonate ramps, SQ1–3 turns to rimmed carbonate platform, and its stratigraphic thickness and sedimentary facies are distributed in NW–SE direction. The succession development of carbonate platform in SQ2 sedimentary period.
(2) The Permian tectonic–sedimentary differentiation in northeastern Sichuan Basin began in the early stage of the Middle Permian. To be specific, from the early to late stage of the Maokou Formation, deep-water sediments dominated by platform-basin facies gradually expanded in the SW–NE direction towards the basin interior, and eventually a sedimentary pattern with platform in the south and platform-basin in the north was formed. The N-E tensional stress generated by the expansion of the Mianlue Ocean is the main contributor to the tectonic–sedimentary differentiation in northeastern Sichuan Basin.
(3) The platform–trough sedimentary landform pattern formed by the tectonic–sedimentary differentiation makes the Maokou Formation in northeastern Sichuan Basin a prospect for both conventional and unconventional oil and gas exploration.

## Supporting information

S1 File This manuscript provides the sequence strate thickness of the wells and outcrops of the Maokou Formation in northeastern Sichuan Basin (supplementary tables). (PDF)
