# Supplementary material for: Sequence–lithofacies paleogeographic evolution and its control on deep and ultra–deep reservoir types: A case study of the Permian Maokou Formation in northeastern Sichuan Basin
Source: PLoS One. 2025 Jul 1;20(7):e0327224. doi: 10.1371/journal.pone.0327224 (PMC12212539; doi:10.1371/journal.pone.0327224)
Supplement: S1 File — (PDF) [file pone.0327224.s001.pdf]

Table S1 Sequence thickness of wells in the northeastern part of the Sichuan Basin

Well	SQ1-1			SQ1-2			SQ1-3			SQ2		
	Top depth(m)	Bottom depth(m)	thickness(m)	Top depth(m)	Bottom depth(m)	thickness(m)	Top depth(m)	Bottom depth(m)	thickness(m)	Top depth(m)	Bottom depth(m)	thickness (m)
QL5	4802.00	4862.50	60.5	4734.20	4802.00	67.8	4679.50	4734.20	54.7	4679.50	4672.16	7.34
QL14	4344.40	4405.20	60.8	4276.40	4344.40	68	4224.40	4276.40	52	4224.40	4193.16	31.24
LB1	5585.94	5669.00	83.06	5547.18	5585.94	38.76	5483.51	5547.18	63.67	5483.51	5455.33	28.18
YH2	4700.20	4756.10	55.9	4630.36	4700.20	69.84	4584.30	4630.36	46.06	4584.30	4515.50	68.8
YH3	4536.76	4594.70	57.94	4470.70	4536.76	66.06	4419.96	4470.70	50.74	4419.96	4366.84	53.12
YH13	4512.12	4567.50	55.38	4448.08	4512.12	64.04	4397.70	4448.08	50.38	4397.70	4334.20	63.5
YA1	2775.32	2847.60	72.28	2665.71	2775.32	109.61	2600.40	2665.71	65.31	2600.40	2596.89	3.51
YA2	5080.36	5141.40	61.04	5020.92	5080.36	59.44	4958.60	5020.92	62.32	4958.60	4947.59	11.01
YA6	4367.55	4433.00	65.45	4310.65	4367.55	56.9	4268.45	4310.65	42.2	4268.45	4259.40	9.05
YA7-1	5570.60	5632.10	61.5	5514.60	5570.60	56	5439.90	5514.60	74.7	5439.90	5434.75	5.15
YA8	4754.28	4813.00	58.72	4685.64	4754.28	68.64	4639.50	4685.64	46.14	4639.50	4621.20	18.3
YA9	2780.80	2841.50	60.7	2718.44	2780.80	62.36	2655.40	2718.44	63.04	2655.40	2649.92	5.48
YA12	5116.48	5180.00	63.52	5057.12	5116.48	59.36	5006.60	5057.12	50.52	5006.60	4981.70	24.9
YA19	5179.70	5248.50	68.8	5123.90	5179.70	55.8	5101.15	5123.90	22.75	5101.15	5092.65	8.5
YA21	5585.04	5650.60	65.56	5524.88	5585.04	60.16	5449.70	5524.88	75.18	5449.70	5440.08	9.62
T4	4961.90	5028.80	66.9	4906.30	4961.90	55.6	4866.40	4906.30	39.9	4866.40	4839.90	26.5
RT1	6111.40	6180.20	68.8	6061.40	6111.40	50	5988.60	6061.40	72.8	5988.60	5984.2	4.4
YB3	7320.70	7379.10	58.4	7264.00	7320.70	56.7	7211.88	7264.00	52.12	7211.88	7144.70	67.18
YB6	7108.30	7167.10	58.8	7069.90	7108.30	38.4	7022.32	7069.90	47.58	7022.32	6947.00	75.32
YB7	7188.80	7248.40	59.6	7149.10	7188.80	39.7	7097.92	7149.10	51.18	7097.92	6942.00	155.92
YB8	7420.05	7464.91	44.86	7378.06	7420.05	41.99	7328.70	7378.06	49.36	7328.70	7185.72	142.98
YB13	6227.55	6283.35	55.8	6188.25	6227.55	39.3	6142.45	6188.25	45.8	6142.45	6090.15	52.3
YB222	7174.50	7210.60	36.1	7132.20	7174.50	42.3	7089.50	7132.20	42.7	7089.50	6999.45	90.05
YB223	7112.70	7152.90	40.2	7057.60	7112.70	55.1	7000.90	7057.60	56.7	7000.90	6915.65	85.25
YB224	7103.90	7149.50	45.6	7064.30	7103.90	39.6	7017.30	7064.30	47	7017.30	6932.30	85
YS1	7092.40	7156.60	64.2	7039.00	7092.40	53.4	7002.4	7039	36.4	7002.4	6884.4	118
LD3	4045.36	4110.50	65.14	3963.20	4045.36	82.16	3899.44	3963.20	63.76	3899.44	3849.80	49.64
LD6	4517.36	4608.00	90.64	4440.40	4517.36	76.96	4376.24	4440.40	64.16	4376.24	4312.70	63.54
JT1	7787.7	7844.95	57.25	7732.08	7787.7	55.62	7680.17	7732.08	51.91	7680.17	7601.69	78.48
JG1	7369.60	7425.29	55.69	7315.85	7369.60	53.75	7252.27	7315.85	73.34	7252.27	7194.57	57.7
W76	4820.36	4882.50	62.14	4742.97	4820.36	77.39	4681.48	4742.97	61.49	4681.48	4616.40	65.08
W80	5259.12	5322.00	62.88	5175.28	5259.12	83.84	5115.04	5175.28	60.24	5115.04	5056.80	58.24
W88	4164.76	4222.90	58.14	4088.58	4164.76	76.18	4033.30	4088.58	55.28	4033.30	3975.50	57.8
W114	4628.3	4691	62.7	4556.8	4628.3	71.5	4516.2	4556.8	40.6	4516.2	4417	99.2
W115	4717.60	4778.00	60.4	4642.68	4717.60	74.92	4591.72	4642.68	50.96	4591.72	4508.50	83.22
ST1	7113.91	7185.25	71.34	6995.68	7113.91	118.23	6929.88	6995.68	65.8	6929.88	6852.75	77.13
ST2	5492.20	5564.20	72	5436.95	5492.20	55.25	5391.8	5436.95	45.15	5391.8	5363.5	28.3
ST3	7350.94	7420.40	69.46	7251.62	7350.94	99.32	7194.53	7251.62	57.09	7194.53	7116.00	78.53
ST6	7712.60	7778.00	65.4	7631.00	7712.60	81.6	7571.2	7631.00	59.8	7571.2	7466.51	104.69

ST7	7514.15	7589.00	74.85	7414.19	7514.15	99.96	7371.96	7414.19	42.23	7371.96	7270.8	101.16
ST8	7215.20	7285.20	70	7113.84	7215.20	101.36	7062.51	7113.84	51.33	7062.51	6945.8	116.71
ST9	7599.36	7670.80	71.44	7516.10	7599.36	83.26	7468.08	7516.10	48.02	7468.08	7383.82	84.26
ST10	7340.97	7416.00	75.03	7250.90	7340.97	90.07	7186.76	7250.9	64.14	7186.76	7092.92	93.84
ST12	6974.85	7047.05	72.2	6871.59	6974.85	103.26	6818.70	6871.59	52.89	6818.70	6725.25	93.45
ST101	7560.05	7654.00	93.95	7465.18	7560.05	94.87	7406.4	7465.18	58.78	7406.4	7307	99.4
SS1	5104.20	5195.64	91.44	5047.64	5104.20	56.56	4988.50	5047.64	59.14	4988.50	4982.00	6.5
WJ1	3812.17	3872.01	59.84	3754.60	3812.17	57.57	3698.83	3754.60	55.67	3698.83	3657.9	40.93
F1	4078.9	4150.5	71.6	4016.28	4078.9	62.62	3926.04	4016.28	90.24	3926.04	3897.08	28.96
F2	4192.2	4273	80.8	4120.48	4192.2	71.72	3986.38	4076.32	89.94	3986.38	3942.1	44.28
DT4	4858.76	4919.60	60.84	4787.72	4858.76	71.04	4736.50	4787.72	51.22	4736.50	4726.92	9.58
DW4	5975.28	6043.12	67.84	5916.85	5975.28	58.43	5886.64	5916.85	30.21	5886.64	5872.25	14.39
TD7	4399.20	4463.50	64.3	4322.88	4399.20	76.32	4271.10	4322.88	51.78	4271.10	4267.60	3.5
TD20	4715.64	4776.40	60.76	4647.56	4715.64	68.08	4593.00	4647.56	54.56	4593.00	4589.75	3.25
TD56	4561.00	4623.40	62.4	4492.92	4561.00	68.08	4450.20	4492.92	42.72	4450.20	4422.30	27.9
TD58	4689.36	4750.00	60.64	4622.48	4689.36	66.88	4574.40	4622.48	48.08	4574.40	4569.52	4.88
TD84	4729.24	4793.10	63.86	4648.52	4729.24	80.72	4573.00	4648.52	75.52	4573.00	4501.20	71.8
TD87	4548.80	4608.68	59.88	4473.90	4548.80	74.9	4421.80	4473.90	52.1	4421.80	4362.00	59.8
TD90	4741.00	4803.00	62	4675.36	4741.00	65.64	4616.80	4675.36	58.56	4616.80	4528.40	88.4
TD94	4688.24	4750.00	61.76	4623.52	4688.24	64.72	4563.28	4623.52	60.24	4563.28	4474.10	89.18
TD99	4442.44	4508.00	65.56	4372.84	4442.44	69.6	4326.50	4372.84	46.34	4326.50	4317.70	8.8
TC1	4905.28	4967.84	62.56	4855.44	4905.28	49.84	4837.20	4855.44	18.24	4837.20	4827.56	9.64
TX1	233.70	308.05	74.35	184.40	233.70	49.3	125.5	184.4	58.9	125.5	112.2	13.3
TX2	4572.68	4637.00	64.32	4504.08	4572.68	68.6	4445.96	4504.08	58.12	4445.96	4392.00	53.96
B2	4646.7	4718.53	71.83	4570.68	4646.7	76.02	4494.6	4570.68	76.08	4494.6	4435.56	59.04
XH1	6143.12	6208.60	65.48	6087.24	6143.12	55.88	6025.00	6087.24	62.24	6025.00	6016.04	8.96
ZG2	3625.20	3695.00	69.8	3563.90	3625.20	61.3	3487.30	3563.90	76.6	3487.30	3481.05	6.25
S1	5376.60	5467.60	91	5326.05	5376.60	50.55	5281.7	5326.05	44.35	5281.7	5235.88	45.82
F7	4876.05	4942.50	66.45	4814.00	4876.05	62.05	4738.50	4814.00	75.5	4738.50	4726.65	11.85
CF85	5229.7	5293	63.3	5160.88	5229.7	68.82	5102.89	5160.88	57.99	5102.89	5089.2	13.69
CY84	5155.1	5222.2	67.1	5096.8	5155.1	58.3	5038.08	5096.8	58.72	5038.08	5029.2	8.88
CS1	6769.48	6825.60	56.12	6702.52	6769.48	66.96	6656.12	6702.52	46.4	6656.12	6565.88	90.24
JS1	3516.2	3585	68.8	3445.6	3516.2	70.6	3363.4	3445.6	82.2	3363.4	3331	32.4
Z12	4668.20	4731.00	62.8	4587.36	4668.20	80.84	4525.92	4587.36	61.44	4525.92	4471.90	54.02
Z17	4533.16	4590.00	56.84	4453.72	4533.16	79.44	4402.92	4453.72	50.8	4402.92	4311.16	91.76
Z18	4368.50	4425.50	57	4289.04	4368.50	79.46	4229.55	4289.04	59.49	4229.55	4163.52	66.03
C23	4225.16	4280.55	55.39	4154.90	4225.16	70.26	4109.32	4154.90	45.58	4109.32	4035.70	73.62
C24	3641.48	3690.80	49.32	3574.60	3641.48	66.88	3525.50	3574.60	49.1	3525.50	3465.80	59.7
BX1	5187.90	5251.00	63.1	5119.10	5187.90	68.8	5053.55	5119.10	65.55	5053.55	4985.45	68.1
FD1	4988.05	5063	74.95	4903.33	4988.05	84.72	4831.73	4903.33	71.6	4831.73	4738.66	93.07
PG5	5877.56	5947.40	69.84	5804.19	5877.56	73.37	5748.10	5804.19	56.09	5748.10	5733.00	15.1
BD12	3682.72	3735.00	52.28	3600.90	3682.72	81.82	3549.92	3600.90	50.98	3549.92	3482.60	67.32
BD13	4387.48	4445.50	58.02	4315.00	4387.48	72.48	4254.52	4315.00	60.48	4254.52	4178.36	76.16

L3	3917.36	3996.50	79.14	3844.00	3917.36	73.36	3787.44	3844.00	56.56	3787.44	3726.60	60.84
L6	2639.96	2700.10	60.14	2571.16	2639.96	68.8	2520.40	2571.16	50.76	2520.40	2477.40	43
L7	4643.50	4707.65	64.15	4573.35	4643.50	70.15	4510.20	4573.16	62.96	4510.20	4447.70	62.5
L8	5088.05	5149.40	61.35	5023.00	5088.05	65.05	4963.64	5023.00	59.36	4963.64	4873.60	90.04
SS1	5532.00	5595.90	63.9	5466.20	5532.00	65.8	5418.08	5466.20	48.12	5418.08	5400.24	17.84
C11	2899.85	2967.50	67.65	2831.25	2899.85	68.6	2757.55	2831.25	73.7	2757.55	2687.90	69.65
C16	3213.85	3280.45	66.6	3154.55	3213.85	59.3	3072.80	3154.55	81.75	3072.80	3051.00	21.8
C19	3271.00	3338.80	67.8	3187.40	3271.00	83.6	3116.90	3187.40	70.5	3116.90	3040.40	76.5
C21	3140.65	3206.00	65.35	3068.00	3140.65	72.65	2996.30	3068.00	71.7	2996.30	2961.56	34.74
C25	3059.45	3127.00	67.55	2986.30	3059.45	73.15	2911.95	2986.30	74.35	2911.95	2835.00	76.95
C34	2905.60	2998.50	92.9	2822.10	2905.60	83.5	2747.35	2822.10	74.75	2747.35	2670.80	76.55
C39-1	2945.05	3036.80	91.75	2850.70	2945.05	94.35	2746.30	2850.70	104.4	2746.30	2654.40	91.9
C41	4902.93	4965.50	62.57	4820.50	4902.93	82.43	4749.95	4820.50	70.55	4749.95	4664.80	85.15
C54	2631.00	2700.20	69.2	2564.25	2631.00	66.75	2488.20	2564.25	76.05	2488.20	2427.30	60.9
C61	4157.00	4231.20	74.2	4079.70	4157.00	77.3	4002.70	4079.70	77	4002.70	3933.40	69.3
HB1	5832.20	5902.00	69.8	5755.20	5832.20	77	5717.00	5755.20	38.2	5717.00	5708.00	9
YD1	4933.68	5005.20	71.52	4856.88	4933.68	76.8	4778.64	4856.88	78.24	4778.64	4695.84	82.8
YD2	5259.72	5328.02	68.3	5187.30	5259.72	72.42	5111.96	5187.30	75.34	5111.96	5037.24	74.72
D2	5206.88	5290.80	83.92	5150.48	5206.88	56.4	5112.00	5150.48	38.48	5112.00	5094.76	17.24
D4	5033.24	5095.40	62.16	4982.40	5033.24	50.84	4945.80	4982.40	36.6	4945.80	4930.36	15.44
WQ5	4558.60	4610.95	52.35	4484.40	4558.60	74.2	4441.30	4484.40	43.1	4441.30	4430.52	10.78
K2	2314.96	2402.50	87.54	2244.32	2314.96	70.64	2210.48	2244.32	33.84	2210.48	2163.04	47.44
K3	3465.76	3539.50	73.74	3410.20	3465.76	55.56	3391.60	3410.20	18.6	3391.60	3363.28	28.32
B1	5980.62	6048.71	68.09	5928.87	5980.62	51.75	5866.87	5928.87	62	5866.87	5861.52	5.35
G25	4188.08	4250.95	62.87	4120.40	4188.08	67.68	4062.30	4120.40	58.1	4062.30	4048.40	13.9
G31	5028.64	5097.80	69.16	4958.80	5028.64	69.84	4900.20	4958.80	58.6	4900.20	4890.52	9.68
G1	3503.1	3570.75	67.65	3433.9	3503.1	69.2	3362.96	3433.9	70.94	3362.96	3281.6	81.36
GX1	4755.9	4822.95	67.05	4649.9	4755.9	106	4561.5	4649.9	88.4	4561.5	4446.4	115.1
CZ1	5340	5410	70	5270.9	5340	69.1	5192.96	5270.9	77.94	5192.96	5183.9	9.06
LB3	3536.32	3588.00	51.68	3467.20	3536.32	69.12	3403.60	3467.20	63.6	3403.60	3352.20	51.4
LB5	3630.24	3677.50	47.26	3563.36	3630.24	66.88	3505.70	3563.36	57.66	3505.70	3450.68	55.02
JX2	7026.45	7102.44	75.99	6986.55	7026.45	39.9	6907.05	6986.55	79.5	6907.05	6836.40	70.65
JJ1	4504.35	4567.55	63.2	4426.60	4504.35	77.75	4371.50	4426.60	55.1	4371.50	4308.70	62.8
TS6	3750.05	3822.75	72.7	3676.40	3750.05	73.65	3622.20	3676.40	54.2	3622.20	3614.23	7.97
TS7	4387.40	4462.12	74.72	4305.30	4387.40	82.1	4234.65	4305.30	70.65	4234.65	4227.32	7.33
TS8	3861.68	3928.20	66.52	3795.20	3861.68	66.48	3741.70	3795.20	53.5	3741.70	3723.70	18
MN1	4297.80	4362.20	64.4	4245.40	4297.80	52.4	4206.20	4245.40	39.2	4206.20	4197.91	8.29
MX1	4738.12	4797.00	58.88	4675.80	4738.12	62.32	4616.65	4675.80	59.15	4616.65	4614.05	2.6
MX3	4907.68	4966.80	59.12	4843.60	4907.68	64.08	4787.65	4843.60	55.95	4787.65	4784.90	2.75
MX5	4797.30	4860.90	63.6	4724.40	4797.30	72.9	4671.40	4724.40	53	4671.40	4669.00	2.4
LZ1	6618.3	6678.9	60.6	6544.9	6618.3	73.4	6498.35	6544.9	46.55	6498.35	6416.45	81.9
CJ1	5100.56	5169.00	68.44	5033.76	5100.56	66.8	4969.70	5033.76	64.06	4969.70	4960.50	9.2
L6	3435.44	3503.70	68.26	3367.84	3435.44	67.6	3310.80	3367.84	57.04	3310.80	3303.76	7.04

LX1	4939.64	5004.80	65.16	4880.80	4939.64	58.84	4823.70	4880.80	57.1	4823.70	4818.65	5.05
MC1	3531.40	3632.00	100.6	3483.56	3531.40	47.84	3472.00	3483.56	11.56	3472.00	3467.68	4.32
MC2	3346.00	3417.60	71.6	3298.40	3346.00	47.6	3293.60	3298.40	4.8	3293.60	3285.36	8.24
MS1	6012.80	6084.00	71.2	5968.30	6012.80	44.5	5925.10	5968.30	43.2	5925.10	5906.70	18.4
HL2	4504.30	4562.60	58.3	4435.72	4504.30	68.58	4391.70	4435.72	44.02	4391.70	4380.44	11.26
HL5	5004.20	5070.60	66.4	4950.52	5004.20	53.68	4909.72	4950.52	40.8	4909.72	4892.60	17.12
L17	5772.20	5846.20	74	5715.40	5772.20	56.8	5654.31	5715.40	61.09	5654.31	5646.39	7.92
LH1	4865.32	4958.20	92.88	4765.00	4865.32	100.32	4699.60	4765.00	65.4	4699.60	4681.90	17.7
LT1	5807.50	5872.00	64.5	5745.30	5807.50	62.2	5710.20	5745.30	35.1	5710.20	5692.98	17.22
LT2	6149.80	6214.50	64.7	6089.50	6149.80	60.3	6054.00	6089.50	35.5	6054.00	6026.70	27.3
F1	3751.65	3817.6	65.95	3705.2	3751.65	46.45	3689.4	3705.2	15.8	3689.4	3687.1	2.3
LT1	3093.2	3164	70.8	3047.04	3093.2	46.16	3043.12	3047.04	3.92	3043.12	3028.48	14.64
L1	2748.2	2842	93.8	2695.84	2748.2	52.36	2692.72	2695.84	3.12	2692.72	2675.76	16.96
M5	4447.6	4513.05	65.45	4391.8	4447.6	55.8	4388.6	4391.8	3.2	4388.6	4380.92	7.68
DX5	4008.28	4067	58.72	3958.6	4008.28	49.68	3919.2	3958.6	39.4	3919.2	3908.05	11.15
MB3	5286.1	5352.92	66.82	5226.99	5286.1	59.11	5199	5226.99	27.99	5199	5185.25	13.75
F1	4326.08	4398.32	72.24	4255.44	4326.08	70.64	4171.04	4255.44	84.4	4171.04	4150.92	20.12
SF1	4894.82	4966.71	71.89	4809.28	4894.82	85.54	4739.8	4809.28	69.48	4739.8	4650.54	89.26
HJ1	2990.9	3062.09	71.19	2907.56	2990.9	83.34	2845.7	2907.56	61.86	2845.7	2759.25	86.45
L2	3599	3685.9	86.9	3509.8	3599	89.2	3427.3	3509.8	82.5	3427.3	3389.7	37.6
L5	4787.84	4848.24	60.4	4719.7	4787.84	68.14	4663.9	4719.7	55.8	4663.9	4658.05	5.85
YT1	3450	3510.44	60.44	3402.5	3450	47.5	3388.95	3402.5	13.55	3388.95	3381.1	7.85
HS1	3530.97	3600.4	69.43	3471.54	3530.97	59.43	3424.91	3471.54	46.63	3424.91	3417.55	7.36
YingTan 1	6553	6612	59	6495	6553	58	6443	6495	52	6382	6443	61

Table S2 Sequence thickness of outcrops in the northeastern part of the Sichuan Basin

outcrop	SQ1-1			SQ1-2			SQ1-3			SQ2		
	Top depth(m)	Bottom depth(m)	thickness(m)	Top depth(m)	Bottom depth(m)	thickness(m)	Top depth(m)	Bottom depth(m)	thickness(m)	Top depth(m)	Bottom depth(m)	thickness(m)
Changjianggou	241.60	294.80	53.2	212.70	241.60	28.9	169.60	212.70	43.1	169.60	113.50	56.1
Lengshuixi	986.68	1060.12	73.44	923.56	986.68	63.12	851.75	923.56	71.81	851.75	803.76	47.99
Xueyudong	290.4	352.7	62.3	208.3	290.4	82.1	139.9	208.3	68.4	139.9	3	136.9
Shuanghekou	62.95	116.15	53.2	23.32	62.95	39.63	18.48	23.32	4.84	18.48	9.48	9
Zhengyuan	148.9	202.9	54	90.25	148.9	58.65	28.8	90.25	61.45	28.8	20.21	8.59
Erya	205.15	255.6	50.45	136.2	205.15	68.95	92.56	136.2	43.64	92.56	10	82.56
